# Neurocysticercosis in Low- and Middle-Income Countries, a Diagnostic Challenge from Oyam District, Uganda

**DOI:** 10.3390/idr14040054

**Published:** 2022-07-01

**Authors:** Francesco Vladimiro Segala, Elda De Vita, James Amone, Daniel Ongaro, Ritah Nassali, Bonny Oceng, Samuel Okori, Giovanni Putoto, Peter Lochoro, Jerry Ictho, Massimo Fantoni, Annalisa Saracino, Francesco Di Gennaro

**Affiliations:** 1 Clinic of Infectious Diseases, Catholic University of the Sacred Heart, 00168 Rome, Italy; fvsegala@gmail.com (F.V.S.); massimo.fantoni@policlinicogemelli.it (M.F.); 2 Department of Biomedical Sciences and Human Oncology, Clinic of Infectious Diseases, University of Bari, 70123 Bari, Italy; devitaelda@gmail.com (E.D.V.); annalisa.saracino@uniba.it (A.S.); 3 St. John’s XXIII Hospital Aber, Jaber 21310, Uganda; jamnet5@gmail.com (J.A.); ongarodanieldan@gmail.com (D.O.); ritahnassali29@gmail.com (R.N.); omegabonny@gmail.com (B.O.); drokori@gmail.com (S.O.); 4 Operational Research Unit, Doctors with Africa CUAMM, 35100 Padua, Italy; g.putoto@cuamm.org; 5 Doctors with Africa CUAMM, Kampala 21310, Uganda; p.lochoro@cuamm.org (P.L.); j.ictho@cuamm.org (J.I.)

**Keywords:** Uganda, neurocysticercosis, zoonosis, LMIC, *Tenia solium*, computed tomography

## Abstract

Background: In countries where *Taenia solium* is endemic, neurocysticercosis (NCC) is the leading identified cause of seizures, accounting for nearly 30% of all epilepsy cases and up to 2.8 million of Disability Adjusted Life Years. Diagnosis of this condition, however, is strictly reliant on either MRI or CT scan, which are poorly available in low- and middle-income countries (LMICs), creating challenges for proper case management and the acquisition of precise neuroepidemiologic data that may guide program and policy development. Methods: Here, we report the case of a 73-year-old woman admitted in a rural hospital in Northern Uganda, who presented with seizures and a progressive inability to walk. She was then diagnosed with NCC after a brain CT scan. Conclusions: This case study represents a rare example of the detection of NCC in a rural district hospital, thus suggesting the potential feasibility of a CT-scan guided diagnostic approach in low resource settings.

## 1. Introduction

Cysticercosis is a parasitic zoonosis of both humans and pigs caused by the larval stages of the cestode, a pig tapeworm, *Taenia solium.* [1]. NCC is listed by the World Health Organization (WHO) as one of the neglected tropical diseases, a group of pathologies that affect more than 1 billion people living in tropical areas [1].

In the life cycle of *T. solium*, humans are the definitive host, while swine are the intermediate host [2]. However, after ingesting infected eggs, humans can also act as intermediate hosts. More specifically, cysticercosis is acquired by humans after the ingestion of eggs by the fecal-oral route, e.g., caused by poor hand hygiene, not by eating undercooked pork that contains cysticerci, which is linked to intestinal taeniasis. Autoinfection may occur in humans if proglottids pass from the intestine to the stomach via reverse peristalsis [2]. In these cases, oncospheres hatch in the colon, infiltrate the intestinal wall, enter the blood circulation, and move to different tissues and organs where they mature into cysticerci within 3 months (typically 60–70 days) after the infection [2,3]. 

The lungs, liver, skin, subcutaneous tissues, heart muscle and other tissues, including the oral mucosa, can be invaded by cysticerci. Some cysticerci will migrate to the brain, causing NCC with potentially fatal consequences [3]. NCC represents the greatest burden of *T. solium*-induced disease, which is estimated to contribute to approximately 30% of epilepsy cases in areas where the parasite is endemic.

In 2010, worldwide, NCC resulted in more than 370,000 NCC-associated epilepsy cases, 28,000 deaths and 2.79 million Disability Adjusted Life Years (DALYs), a loss of the equivalent of one year of full health [4]. In eastern Africa, the disease has been reported in Tanzania, Kenya, Uganda, Burundi and Rwanda, and it is believed to be largely underestimated [5]. In Uganda, since the civil war, the establishment of piggeries and increased pig production by rural farmers has been encouraged as part of central government agricultural planning [6]. Pigs are considered low-input livestock, which can easily grow with minimal feeds, and local governments supply piglets to rural families to promote an alternative source of income. In a study conducted in 2009 in the Kamuli and Kaliro districts on 480 pigs, 8.5% were seropositive for the parasite of *Taenia solium* by B158/B60 Ag-ELISA [7].

## 2. Case Presentation

**Figure 1:**
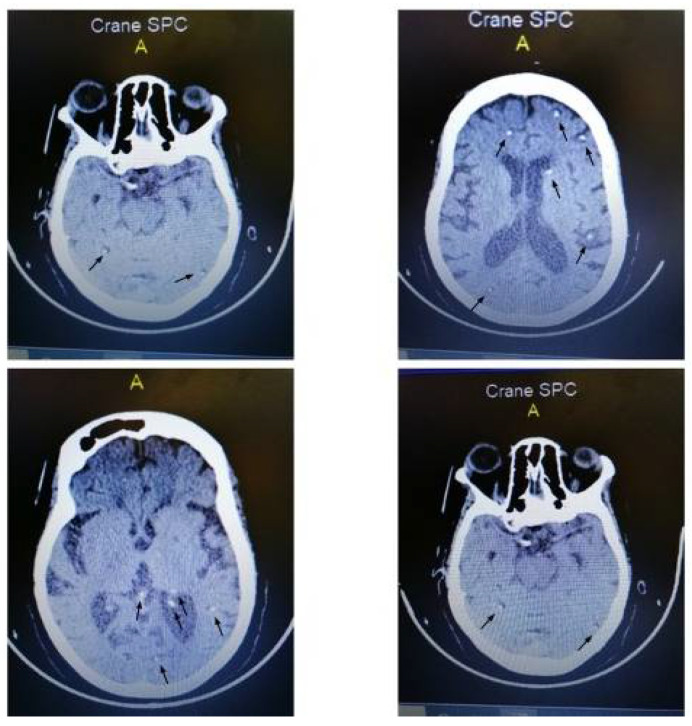
Brain CT scan showing multiple, randomly distributed, round cystic lesions with white dot sign of 4–8 mm in diameter.

Here, we report the case of a 73-year-old woman suffering from high blood pressure and living in the Oyam district of Uganda, who presented in April 2022 to Anyeke level III Health Center with fever, tonic-clonic seizures and the progressive inability to walk, which started one day before. Anti-hypertensive and broad-spectrum antibiotic therapy were started and, three days later, the patient was referred to St. John XIII Hospital of Aber for further management. Here, she presented with severe headache, altered mentation and urine incontinence. On neurological examination, she showed neck stiffness, generalized hyperreflexia and lower limbs hypotonia, with preserved sensory function. She did not present focal neurological deficit and the pupils were hysochoric and normoreactive to light. A routine laboratory test showed no significant abnormalities, while HIV test and thick blood smears for malaria were negative. At hospital admission, lumbar puncture was performed, but cerebrospinal fluid analysis was inconclusive, with normal cell count, glucose, and protein levels. Therefore, a brain CT scan was conducted, which showed signs of multiple round heterodense cystic lesions with white dots, ranging from 4 mm to 8 mm in diameter (Figure 1). Lesions were randomly distributed in the entire supratentorial brain parenchyma, with no associated mass effect or perilesional edema. 

A serological test for *T. solium* was not performed due to a lack of equipment at the hospital.

Based on the radiological features, a presumptive diagnosis of NCC was conducted and the patient was treated with albendazole 15 mg/kg/day and Desamethasone 0.1 mg/kg/day for 10 days. Due to a lack of supplies, Praziquantel 50 mg/kg was added on the third day after diagnosis and administered only for four days. Shortly after treatment was started, the patient showed signs of improvement, re-acquiring alertness and orientation on day 2 and the ability to walk on day 5. She did not develop new episodes of seizures. She was discharged seven days after treatment initiation.

## 3. Discussion

In LMICs (Low- and Middle-Income Countries), while there has been a significant improvement in diagnostic support for HIV/AIDS, tuberculosis, and malaria, significant gaps persists in the availability and quality of diagnostic services for several tropical diseases, even in the case of conditions of public health priority. In LMICs where *T*. *solium* is endemic, NCC is the leading identified cause of seizures [8], while in Uganda, only in 2010, NCC was estimated to be the cause of 9000 new cases of epilepsy and nearly 3000 deaths, leading to an economic burden as high as 8000 USD per NCC case [9]. However, in LMICs, diagnostic tools and technical skills to diagnose this condition are largely unavailable, and only a minimum share of infections are properly identified.

Neuroimaging with either computed tomography scan (CT) or magnetic resonance imaging (MRI) is considered the gold standard for the diagnosis of NCC [10]. Although a CT scan is less sensitive than MRI in identifying ventricular or cisternal cysts [11], its diagnostic performance for intraparenchymal lesions is comparable to that of MRI and is even superior in the presence of calcified lesions [12], with the additional advantage of requiring less technical skills in both interpretations and maintenance, being less expensive to run and generally more available in low resource settings [13]. Moreover, when combined with physical examination and clinical history, the specificity and sensitivity of CT-scans may rise up to, respectively, 99.5% and 98.9% for a single enhancing lesion [14].

In high income countries, where the socio-economic burden of the disease is incomparably lower than that estimated for LMICs, the use and availability of neuroimaging is widespread, and this inequality is further exacerbated by the lack of low-cost, point-of-care diagnostic tests. In a survey conducted by Yadav et al. [15], the most available diagnostic tools were determined to be point-of-care testing for HIV, malaria, viral hepatitis and syphilis, whereas radiologic imaging was among the least available.

In particular, neuroimaging ranks as one of the most unavailable diagnostic tools in LMICs, creating challenges for the proper recognition of several neurologic conditions and impeding the acquisition of precise neuroepidemiologic data for program and policy development [16]. In a study conducted in 10 LMIC in 2021, Uganda was shown to have the third lowest country-level availability of laboratory medicine and imaging diagnostics, with an overall availability of 34.4% [10]. However, in this study, the in-hospital availability of a CT scan was below 20% in all included countries. New perspectives on diagnosis came from a very recent paper defining the role of recombinant monoclonal-based *Taenia* antigen. From this study, the effectiveness of therapy may be monitored in the cerebrospinal fluid (CSF), serum/plasma, and urine using a recently created recombinant monoclonal antibody-based Ts Ag detection ELISA, with high sensitivity in the detection of extra-parenchymal NCC. This method could be crucial in NCC control and diagnosis, especially in low resource settings where the possibility of CT scan or MRI is rare [17].

## 4. Conclusions

Data are lacking regarding the exact incidence of NCC, but our case represents a rare example of the detection of this condition in a rural district Hospital, thus suggesting the potential feasibility of a CT-scan guided diagnostic approach in low resource settings. High quality, longitudinal studies are needed to explore the cost-effectiveness of such an approach in patients with a high index of suspicion for NCC.

## Data Availability

No appliable.
